# In Vitro Osteo-Immunological Responses of Bioactive Calcium Phosphate-Containing Urethane Dimethacrylate-Based Composites: A Potential Alternative to Poly(methyl methacrylate) Bone Cement

**DOI:** 10.1021/acsmaterialsau.4c00037

**Published:** 2024-07-18

**Authors:** Weerachai Singhatanadgit, Piyarat Sungkhaphan, Boonlom Thavornyutikarn, Setthawut Kitpakornsanti, Anne Young, Wanida Janvikul

**Affiliations:** † Faculty of Dentistry and Research Unit in Mineralized Tissue Reconstruction, Thammasat University (Rangsit Campus), Pathum-thani 12121, Thailand; ‡ National Metal and Materials Technology Center, National Science and Technology Development Agency, Khlong Luang 12120, Thailand; § Division of Biomaterials & Tissue Engineering, UCL Eastman Dental Institute, Royal Free Hospital, Hampstead, London NW3 2PF, U.K.

**Keywords:** osteo-immunological response, monomer conversion, particle distribution, monocalcium phosphate, hydroxyapatite, urethane dimethacrylate-based composite cements

## Abstract

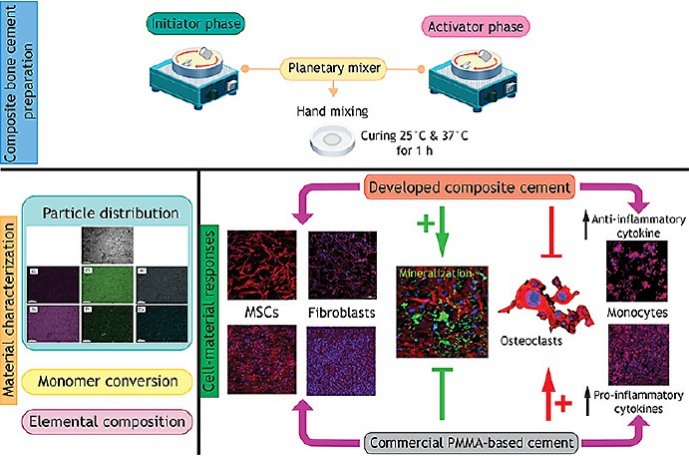

This investigation developed new composite bone cements using urethane dimethacrylate (UDMA), poly(propylene glycol) dimethacrylate (PPGDMA), and hydroxyethyl methacrylate (HEMA), with micrometer-sized aluminosilicate glass filler. Monocalcium phosphate monohydrate (MCPM) and hydroxyapatite (HA) particles were added to enhance biological performance, particularly osteo-immunomodulation. Free radical polymerization was triggered by mixing two pastes containing either benzoyl peroxide (BPO, an initiator) or N-tolyglycine glycidyl methacrylate (NTGGMA, an activator). Increasing butylated hydroxytoluene (BHT, an inhibitor) enabled a suitable delay after mixing at 25 °C for placement. At 37 °C, the delay time was reduced and the final conversion was enhanced. Findings also demonstrated the biocompatibility of the developed bone cement toward osteo-immunological cell lineages, including mesenchymal stem cells (MSCs), fibroblasts, osteoclast precursor RAW 246.7 cells, and peripheral blood mononuclear cells (PBMCs). Notably, the cement with both MCPM and HA combined facilitated sufficient MSC growth, enabling subsequent mineralization while concurrently suppressing the proliferation of fibroblasts, osteoclast progenitors, and PBMCs. Furthermore, composite cement exhibited the capacity to differentially regulate osteoblast differentiation, cell-(in)dependent mineralization, osteoclastogenesis, and PBMC-mediated inflammatory responses at both cellular and molecular levels in vitro. These observations suggested their potential use for bone repair, especially in cases of inflammation-associated bone defects.

## 1 Introduction

Bone cements must be biocompatible and noncytotoxic to surrounding bone and immune cells, facilitating physiological crosstalk between these cells during normal bone healing. Ideally, these materials should support the attachment, development, and differentiation of mesenchymal stem cells (MSCs) into mature bone-producing osteoblasts, without causing fibrous tissue formation, prolonged inflammatory cytokine expression, or excessive osteoclast development.^1^ Poly(methyl methacrylate) (PMMA) has been the most known and widely used bone cement in various medical applications, particularly in orthopedic and cranio-maxillo facial surgery and traumatology.^2^ It has been used for osteoporotic vertebroplasty, fracture fixation, and craniofacial bone repair.^2,3^ However, this material has biologically related disadvantages, including high heat of polymerization and release of toxic monomer residues.^2^ Exothermic reactions can unfavorably cause necrosis of the surrounding cells and tissues, and residual monomer molecules may induce excessive inflammatory responses and osteoclastic bone destruction. These shortcomings may be mitigated using higher molecular weight dimethacrylate monomers such as urethane dimethacrylate (UDMA) that is widely used in dental composites^4^ and oligomers such as poly(propylene glycol) dimethacrylate (PPGDMA).^4−6^

Many studies have investigated improvements in the biological activity of PMMA cements by adding calcium phosphates (CaPs), providing the essential ions for the formation of such inorganic bone components as hydroxyapatite (HA). HA is one of the most studied CaP additives incorporated into PMMA bone cements. Researchers have found that HA addition enhances not only the physical and mechanical properties^7^ but also the biocompatibility and osteogenic potential of PMMA cements.^8−12^ These benefits might be associated with their remarkable ability to interact with cells. It stimulated the expression of genes responsible for bone matrix formation in preosteoblastic cells^13^ and induced uncommitted cells to differentiate into the osteoblast lineage.^14^ Furthermore, its effects on osteoblastic lineage and osteoclast formation and activity have been shown to be controlled by its nanograin and submicrograin structures.^15^ HA also modulated inflammatory responses, depending on the characteristics of HA particles.^16,17^ For example, nanosized and small microsized HA particles (<20 μm) significantly boosted pro-inflammatory cytokine secretion with a considerably reduced capacity to secrete interleukin (IL)-10, a potent anti-inflammatory cytokine, while larger microsized particles of approximately 100 μm allowed normal production of IL-10.^16^ Moreover, HA-containing composites appeared immunologically inert^18^ and biocompatible, eliciting no significant alterations in hematological, serum biochemical, or histopathological parameters in vivo.^19^ It is thus possible that the incorporation of large HA microparticles into bone composites may induce new bone formation and provide an opportunity to fine-tune the host response by directing the initial pro-inflammatory immune response toward a bone regenerative microenvironment following implantation. However, one potential drawback of using HA in methacrylate-based bone cements is its limited solubility or release from the material. This can hinder the expected biological activity of HA and potentially compromise the clinical application of such cements.

Unlike HA, monocalcium phosphate (MCP) is more soluble and acidic and can be released from methacrylates. Under the acidic conditions present during the inflammatory phase of bone repair, released MCP ions are expected to interact with the more basic HA to form other calcium phosphates such as dicalcium phosphate (brushite). This brushite is then expected to transform into HA in the latter, neutral, and calcium-rich phases of bone repair. MCP can promote surface mineral precipitation on methacrylate-based composites in simulated body fluid.^20−23^ Previous studies have shown that MCP-containing UDMA-based composites possessed mineralized tissue-compatible mechanical strength and self-healing potential.^4−6^ Compared to commercial bone cement products (Cortoss and Simplex P), the chemical-activated MCP-containing UDMA-based bone composites had comparable monomer conversions (%) and flexural strength.^5^ Newly developed UDMA-based dental composites containing bioactive MCP provided an etching effect and good adaptation to mineralized tissues, such as enamel and dentine,^6^ which have similar compositions to bone. Although these favorable properties make MCP a good candidate as a CaP additive in bone cements, the biological activity of MCP-incorporated methacrylate-based bone cement remains largely unexplored.

In this study, UDMA-based bone cement pastes, containing micrometer-sized CaPs (i.e., MCPM and/or HA) and a polymerization inhibitor to obtain suitable working times, were formulated at different compositions. The monomer conversion (%) as a function of curing time of each cement composite was determined by Attenuated Total Reflection-Fourier Transform Infrared spectroscopy (ATR-FTIR), and the distributions of the inorganic particles, i.e., aluminosilicate glasses, MCPM and HA, integrated in the composite were simultaneously analyzed by scanning electron microscopy equipped with energy dispersive X-ray analysis. Their osteo-immunomodulatory roles were also evaluated based on previously reported in vitro models, using cultures of specific cell types known to be involved in bone repair.^24−26^

## 2 Materials and Methods

### 2.1 Materials

All chemicals used were of analytical grade: UDMA (a base monomer) (Rahn AG, Switzerland, *M*_w_ = 470), PPGDMA (a diluent monomer) (Polysciences Inc., USA, *M*_w_ = 560), hydroxyethyl methacrylate (HEMA, a diluent monomer) (Sigma-Aldrich, St. Louis, USA), benzoyl peroxide (BPO, an initiator) (Sigma-Aldrich), N-tolyglycine glycidyl methacrylate (NTGGMA, an activator) (Esstech Inc., USA), butylated hydroxytoluene (BHT, an inhibitor) (Sigma-Aldrich), monocalcium phosphate monohydrate (MCPM, a bioactive filler, average particle size = 53 μm) (Himed, Old Bethpage, USA), HA (a bioactive filler, average particle diameter = 5–20 μm) (Taihei Chemical Industrial Co., Ltd., Osaka, Japan), and silane-treated aluminosilicate glass particles (fillers, average diameters = 0.7 and 7 μm) (DMG, Hamburg, Germany).

### 2.2 Bone Cement Preparation

#### 2.2.1 Bone Cement Paste Preparation

UDMA-based composites (CPs) were prepared by mixing initiator and activator pastes. Each paste contained liquid (i.e., monomers) and powder (i.e., glass + MCPM + HA) phases.

For liquid phases, UDMA and PPGDMA were used at a fixed weight ratio of 2:1 and then HEMA was added at 2.5 wt %. Once homogeneous, BHT (0.28–0.56 wt %) and BPO (1.5 wt %) were combined to give an initiator liquid, while NTGGMA (1 wt %) was mixed into the activator liquid. Each liquid phase was stirred at room temperature for 2 h, followed by sonication at 40 °C for 2 h to ensure the complete dissolution of the solid particles.

To produce pastes, aluminosilicate glass (average diameters of 7 and 0.7 μm), combined at a weight ratio of 1:1, was integrated into both initiator and activator liquids. MCPM (5.6 or 6.0 wt % of whole CP) and/or HA (5.6 wt % of whole CP) were/was added in the initiator paste (Table 1). The ratios of total powder to liquid for CP1-CP2 were 2.6:1 in both the initiator and activator pastes. For CP3-CP5, this was 3:1. For CP6-CP8, powder:liquid was 3:1 for the activator paste but reduced slightly to 2.3:1 for the initiator paste. Varying this ratio enabled more consistent paste rheological characteristics and ease of mixing when HA was present (Table S1 in the Supporting Information). The thorough mixing of the liquid and powder phases was performed by using a planetary mixer deaerator (Mazerustar, KK-250S, Kurabo, Japan) at a rotation speed of 4300 rpm for 8 min.

**Table 1:** Amounts of MCPM, HA, and BHT Integrated into Each Formulated Composite Paste

composite pastes	CaPs (%wt of whole composite paste)		BHT (%wt of whole monomer mixture)
	MCPM	HA	
CP1	0	5.6	0
CP2	0	5.6	0.28
CP3	0	0	0
CP4	0	0	0.52
CP5	6.0	0	0.52
CP6	0	5.6	0.56
CP7	5.6	5.6	0.56
CP8	5.6	5.6	0.28

#### 2.2.2 Bone Cement Disc Preparation

To fabricate composite discs, the initiator and activator pastes for each formulated cement were mixed at a 1:1 weight ratio on a paper pad for 1 min, placed in a metal circlip (10 mm internal diameter and 1 mm thickness) used as a mold, and subsequently set in an oven at 37 °C for 1 h. Once cured, the composite discs were removed from the mold; any overflowed material was trimmed, and the prepared discs were UV sterilized before being used in cell studies.

### 2.3 Monomer Conversion

The degree of (monomer) conversion affects both the durability of the restoration and physical and biological properties of materials.^22,27,28^ Kinetics of composite polymerization was analyzed by ATR-FTIR at 25 °C (THERMO/Nicolet 6700 FTIR spectrometer) and/or 37 °C (PerkinElmer series 2000 FTIR spectrometer). Briefly, equal masses of initiator/activator pastes were mixed and then immediately placed in a metal circlip centered on the diamond ATR crystal. The top surface of the paste was covered with an acetate sheet to prevent surface oxygenation inhibition of the polymerization reaction. The FTIR spectrum in the region of 500–4000 cm^–1^ with a resolution of 4 cm^–1^ on the lower sample surface was recorded for 40 min. Continuous spectral acquisition enabled conversion determination without data normalization. The monomer conversion (MC) at a given time (*t*) after mixing was calculated from the peak heights at 1636 cm^–1^ initially (*H*_i_) and after time *t* (*H_t_*) above background using the following equation:
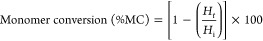
1

### 2.4 Particle Distribution

Scanning electron microscopy equipped with energy dispersive X-ray analysis (SEM/EDX-EBSD (HITACHI/S-3400N, Japan) was employed for the analysis of the composition and spatial distribution of the elements. Surface and cross-sectional areas of fractured composite disc specimens were assessed using an accelerating voltage of 20 kV. Acquisition time to map each square was 200 s with a count rate optimization process time of 6 min. The atomic weight percentages of the organic and inorganic elements detected by EDX analysis in the top surface versus the bulk fracture site were compared.

### 2.5 Cell Culture and Experimental Design

In the present study, the cytocompatibility of bone cements was assessed using MSCs, fibroblasts, osteoclast precursor RAW 264.7 (RAW) cells, and pro-inflammatory peripheral blood mononuclear cells (PBMCs). MSCs and fibroblasts were also used to examine the regulatory role of the tested bone cements in osteoblast-mediated mineralization. Mouse RAW cells were used to determine the materials’ effects on the development of osteoclasts. The inflammatory cells’ cytokine gene expression was also investigated using human PBMCs. For the use of human tissues in this study, the participants were asked to sign informed consent to the use of the tissues for isolation of fibroblasts and PBMCs following the protocol approved by the Ethics Review Subcommittee for Research Involving Human Research Subjects of Thammasat University No. 3 (049/2564) and the Institutional Biosafety Committee Thammasat University (034/2564).

MSCs (Lonza Biologics plc, Cambridge, UK) were maintained in a standard culture medium consisting of α-minimum essential medium (α-MEM) (Gibco Life Technologies Ltd., Paisley, UK) containing 15% fetal calf serum (FCS) (PAA Laboratories, Yeovil, UK) supplemented with 200 IU/mL penicillin, 200 μg/mL streptomycin, and 2 mM l-glutamine (all from Gibco Life Technologies Ltd.) at 37 °C in a humidified atmosphere of 5% CO_2_ in air. Cells between passages 4–6 were used in this study. For the isolation and culture of fibroblasts, periodontally healthy teeth were collected from patients undergoing routine extraction at the Oral Surgery Clinic, Dental Unit, Thammasat University Hospital. The periodontal ligament tissue was explanted to obtain fibroblasts, and the cells were cultured in α-MEM containing 10% FCS, 200 IU/mL penicillin, 200 μg/mL streptomycin, and 2 mM l-glutamine at 37 °C in a humidified atmosphere of 5% CO_2_ in air. The fibroblasts between passages 3–6 were employed. RAW cells were purchased from the American Type Culture Collection (ATCC TIB-71). Cells were cultured in α-MEM supplemented with 10% FBS, 200 IU/mL penicillin, 200 μg/mL streptomycin, 2 mM l-glutamine in an atmosphere of 5% CO2 and 95% humidity at 37 °C. PBMCs were isolated from buffy coats of fully anonymized healthy donors, as previously described.^29^ The buffy coat was diluted 1:1 with phosphate-buffered saline (PBS), layered on Ficoll-Paque PLUS (GE Healthcare Life Sciences, Piscataway, USA), and centrifuged at 800*g* for 40 min at 18 °C. The PBMC interface layer was collected, washed three times with PBS by centrifugation at 800*g* for 10 min at 18 °C, and placed into 75 cm^2^ culture flasks containing 10 mL of Dulbecco’s Modified Eagle Medium (Gibco Life Technologies Ltd.) containing 10% FCS supplemented with 200 IU/mL penicillin, 200 μg/mL streptomycin, and 2 mM l-glutamine (PBMC standard culture medium).

An in vitro cell culture study was used to assess the bone cements’ cytocompatibility and their effects on the functional activity of osteolineage cells (i.e., osteoblasts and osteoclasts) and pro-inflammatory cells (i.e., PBMCs). The experimental design of the cell culture part of the study is detailed below. For the cytocompatibility study, MSCs, fibroblasts, and RAW cells were cultured in the respective standard culture medium at a density of 2 × 10^3^, 2 × 10^3^, and 2 × 10^5^ cells/cm^2^, respectively, on each bone cement disc for the indicated times. Cell morphology and growth were qualitatively assessed using immunocytological staining of actin and confocal fluorescence microscopy. Viable cells were also quantified using cell viability or DNA quantification assays. To determine the osteogenic activity, MSCs were plated at a density of 2 × 10^4^ cells/cm^2^ on each material sample in the MSC standard culture medium. After 48 h in the medium, the sample was incubated in an osteogenic medium (OM), comprising the MSC standard culture medium supplemented with 0.1 mM l-ascorbic acid 2-phosphate and 10 mM β-glycerophosphate followed by ALP activity measurement (at Days 3 and 7), gene expression assay (at Day 7), tetracycline incorporation assay (at Day 21), and ATR-FTIR analysis of the mineral-to-matrix (mineral/matrix) ratio (at Day 21). For osteoclast formation assay, RAW cells were cultured on test materials at a density of 1 × 10^4^ cells/cm^2^ in an osteoclastic medium (OCM), containing the RAW cell standard culture medium supplemented with 50 μg/mL RANKL (PeproTech EC Ltd., London, UK) for 4 days before being subjected to scanning electron microscopy, immunocytological staining of actin, and gene expression assay. For the inflammatory responses to the tested bone cements, PBMCs were cultured at a density of 2 × 10^5^ cells/cm^2^ on bone cement discs in the PBMC standard culture medium supplemented with 200 ng/mL lipopolysccharide (LPS; Sigma-Aldrich) for 6 and 24 h, and the activation of PBMCs was assessed by measuring the DNA content and gene expression assays.

### 2.6 Immunocytological Staining of Actin

To visualize the morphology of cells cultured on bone cement discs, the samples were analyzed by confocal fluorescence microscopy of staining of cellular actin filaments and nuclei. After the indicated times in culture, the samples were fixed with 4% paraformaldehyde (Sigma-Aldrich) and permeabilized with 0.1% Triton X-100, and the prepared samples were stained with rhodamine-phalloidin and DAPI (Invitrogen) following the manufacturer’s instruction. Digital images were obtained under a confocal fluorescence microscope (Nikon Ti Eclipse, Nikon Instruments Inc., NY, USA).

For morphological analysis of MSCs and fibroblasts, cell area, circularity, perimeter, and Feret’s diameter were quantified after 24 h postseeding based on digital images obtained above. For each sample, six images were taken in different areas for all analyses. The digital images were analyzed with NIH ImageJ software (https://imagej.nih.gov/ij/). For quantitative analysis, appropriate thresholding criteria allowed cell areas to be accurately distinguished from those without cell staining. Cells that appeared to be clearly separated were used for further analysis. Data from at least 50 cells were averaged for each sample. For visualization of osteoclasts, the number of osteoclasts (multinucleated cells with more than 2 nuclei) per area (mm^2^) was also counted manually.

### 2.7 Cell Viability Assay

Cell viability was measured by using a trypan blue exclusion assay. Single-cell suspensions prepared by using the dissociation reagent TrypLE Express (Gibco) for 5 min at 37 °C with gentle pipetting were subjected to the test, and the total number of viable cells per surface area of each sample was calculated.

### 2.8 ALP Activity Assay

The ALP activity was measured using histochemical and biochemical analyses, as described previously.^30^ For histochemical analysis, each sample was fixed with 1% paraformaldehyde and then incubated with the 5-bromo-4-chloro-3-indolyl phosphate/nitro blue tetrazolium (BCIP/NBT) liquid substrate (Sigma-Aldrich) at 37 °C. The ALP-positive cells were visualized as dark blue color. For biochemical analysis, cell samples were incubated in radioimmunoprecipitation assay (RIPA) lysis buffer, and an aliquot with an equal amount of protein content was analyzed by incubating with 10 mM *p*-nitrophenylphosphate (*p*-NPP; Sigma-Aldrich) at 37 °C for 10 min. The reaction was then stopped by 3 N NaOH. The amount of the *p*-nitrophenol (*p*-NP) product, corresponding to the ALP activity, was measured at *A*_560_.

### 2.9 Gene Expression Assay

Quantitative real-time PCR (qPCR) was performed to examine the expression of genes associated with osteoblast differentiation (Runt-related transcription factor-2 (RUNX2), type-I collagen (COL-I) and osteocalcin (OCN)), osteoclast differentiation (tartrate-resistant acid phosphatase (TRAP) and cathepsin K (CTSK)) and inflammation (cyclo-oxygenase 2 (COX2), IL-1β, IL-6, tumor necrosis factor (TNF)-α and IL-4) after exposure of cells to each material for indicated times. qPCR using SYBR Green I Master kit (Roche Diagnostic Co.) was performed in an iQ5 iCycler (BioRad, Bradford, UK), with specific primers shown in Table 2.^31,32^ GAPDH was used as an endogenous control. qPCR was performed with six replicates per sample. The mRNA expression levels were normalized to glyceraldehyde-3-phosphate dehydrogenase (GAPDH) expression within each reaction. Fold-change in mRNA expression relative to a control group (set to 1.0) is presented as the mean ± SD of three independent experiments.

**Table 2:** Primer Sequences Used in the Study

genes	forward sequences	reverse sequences
human GAPDH	5′- CTGGGCTACACTGAGCACC-3′	5′- AAGTGGTCGTTGAGGGCAATG-3′
human RUNX2	5′-TGGTTACTGTCATGGCGGGTA-3′	5′-TCTCAGATCGTTGAACCTTGCTA-3′
human COL-I	5′-GAGGGCCAAGACGAAGACATC-3′	5′-CAGATCACGTCATCGCACAAC-3′
human OCN	5′- CACTCCTCGCCCTATTGGC-3′	5′-CCCTCCTGCTTGGACACAAAG-3′
Human COX2	5′- CTGGCGCTCAGCCATACAG-3′	5′- CGCACTTATACTGGTCAAATCCC-3′
human IL-1β	5′-ATGATGGCTTATTACAGTGGCAA-3′	5′-GTCGGAGATTCGTAGCTGGA-3′
human TNF-α	5′-GAGGCCAAGCCCTGGTATG-3′	5′-CGGGCCGATTGATCTCAGC-3′
human IL-4	5′-CTCATTTTCCCTCGGTTTC-3′	5′-GAAGCAGTTGGGAGGTGAG-3′
human IL-6	5′-TTCAATGAGGAGACTTGCC-3′	5′-TGACCAGAAGAAGGAATGA-3′
mouse GAPDH	5′-AGGTCGGTGTGAACGGATTTG-3′	5′- TGTAGACCATGTAGTTGAGGTCA-3′
mouse TRAP	5′- CACTCCCACCCTGAGATTTGT-3′	5′- CATCGTCTGCACGGTTCTG-3′
mouse CTSK	5′- GAAGAAGACTCACCAGAAGCAG-3′	5′- TCCAGGTTATGGGCAGAGATT-3′

### 2.10 Alizarin Red S Staining

Bone cement discs were incubated in the MSC standard culture medium for 3 weeks (with the medium changed every 3 days), and the presence of calcium-containing precipitates (cell-independent mineralization) was assessed by alizarin red S staining.^30,33^ Following staining with a 1% alizarin red S solution, the bone cement discs were rinsed twice with methanol, air-dried, and photographed. To quantify incorporated alizarin red S, a 20 min extraction with 100 mM cetylpyridinium chloride (Sigma-Aldrich) at room temperature was performed. The absorbance of the extracted solution was measured at 570 nm (*A*_570_ nm). Freshly prepared samples were also stained to establish baseline staining levels. These baseline values were then subtracted from the absorbance values obtained from the samples exposed to the culture medium.

### 2.11 Tetracycline Incorporation Assay

For the detection of calcium-containing deposits, tetracycline hydrochloride (Sigma-Aldrich) dissolved in PBS at a final concentration of 20 μg/mL was administrated for 24 h to the cells under osteogenic induction at Days 13 and 20. After 21 days in OM culture, cells were washed twice with PBS, and the samples were stained with rhodamine-phalloidin and DAPI (Invitrogen), as described above. Digital images were taken with a confocal fluorescence microscope (Nikon Ti Eclipse).

### 2.12 ATR-FTIR Spectroscopy

To determine the mineral/matrix ratio of each of the samples harvested from MSC monolayers cultured on test materials in OM for 21 days, ATR-FTIR analysis was carried out using a Nicolet iS 5 FTIR Spectrometer (Thermo Scientific, MA, USA). ATR-FTIR spectra were obtained with a spectral resolution of 4 cm^–1^, and a scan range of 800–1800 cm^–1^. Background subtraction and normalization were undertaken using OMNIC software (Thermo Scientific). Peaks at 1020 and 1650 cm^–1^ were assigned to the phosphate stretching vibration of apatite and the amide I stretching vibration of the bone organic matrix, respectively. Relative mineral/matrix ratios were obtained from the ratio of these peaks.^34^ The mineral/matrix ratios of swine spine cancellous bone (used as a positive control value) and MSCs cultured without OM (used as a baseline control value) were also recorded.

### 2.13 SEM Analysis of Osteoclast Formation

To prepare the samples for imaging, they were initially fixed with 3% glutaraldehyde in a 0.14 M sodium cacodylate buffer solution (pH 7.3) at 4 °C overnight. Subsequently, the samples underwent dehydration through a graded ethanol series (50%, 70%, 90%, and two incubations in 100% ethanol). Following dehydration, the samples were washed with hexamethyldisilazane (TAAB Laboratories, Berkshire, UK) for 5 min. After being air-dried, the samples were sputter-coated with gold for further analysis. Cell morphology was observed by using a JEOL JCM 6000 scanning electron microscope (JEOL UK, Welwyn Garden City, UK). RAW cells cultured on dentine slices in the OCM were used as a positive control.

### 2.14 DNA Content Quantification Assay

After harvesting PBMCs from the test materials, ice-cold ethanol fixed single-cell suspensions were prepared at 1 × 10^6^ cells/mL density in PBS. The samples were stained with 100 μg/mL RNase A and 50 μg/mL propidium iodide (both from Sigma-Aldrich) in PBS for 30 min at room temperature and analyzed under the CytoFLEX benchtop flow cytometer (Beckman Coulter Life Sciences, Indianapolis, USA).

### 2.15 Statistical Analysis

Data are expressed as the mean ± SD from triplicate measurements. Statistical analysis was performed using one-way analysis of variance (ANOVA) with posthoc Dunnett’s test via SPSS software (SPSS, Inc., Chicago, USA). A *p*-value less than 0.05 was considered statistically significant.

## 3 Results and Discussion

### 3.1 Percentage Monomer Conversion

**Figure 1:**
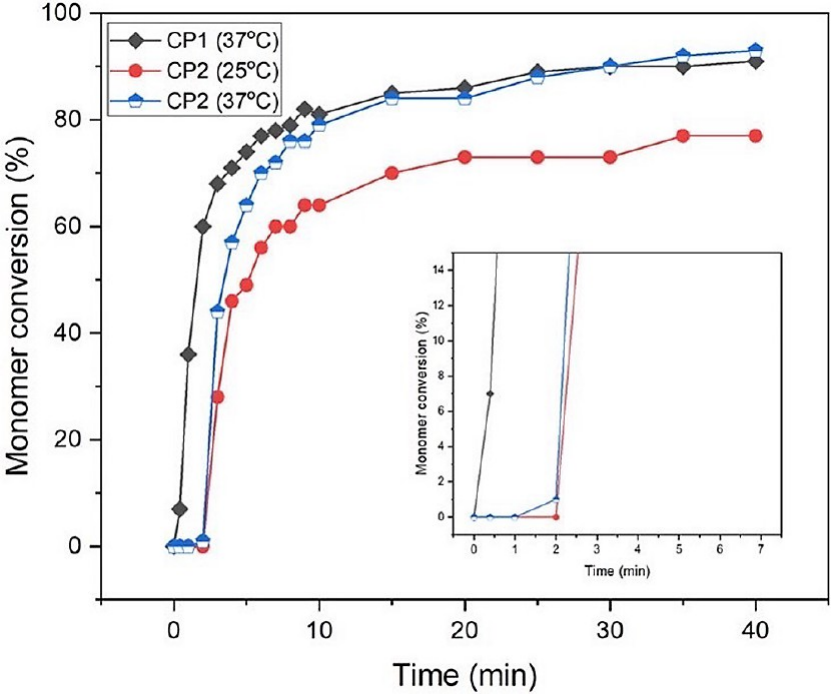
Percentage monomer conversions as a function of polymerization time of CP1–CP2, measured from their ATR-FTIR spectra continuously recorded at 25 °C and/or 37 °C for 40 min.

**Figure 2:**
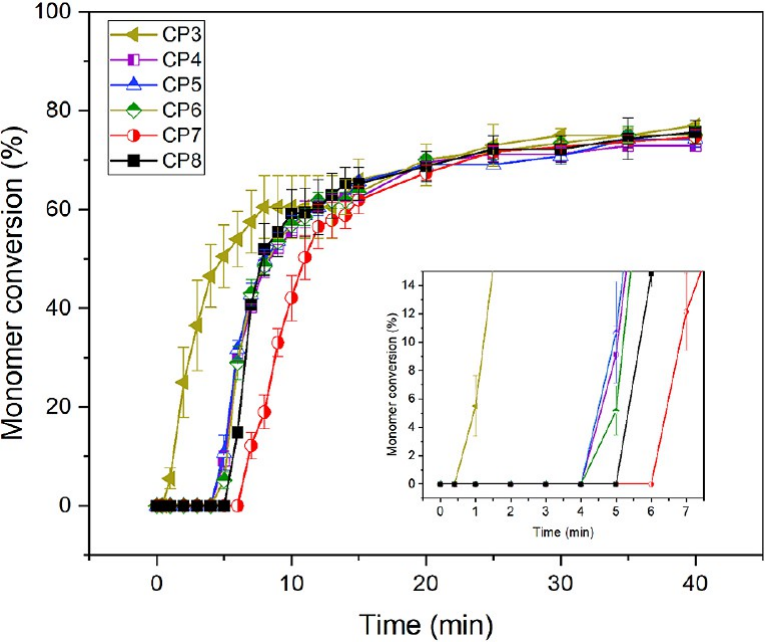
Percentage monomer conversions as a function of polymerization time of individual composite pastes, measured from their ATR-FTIR spectra continuously recorded at 25 °C for 40 min.

Composite spectral changes upon polymerization (see Figure S1) were all comparable with those previously observed.^5^ Examples of composite percentage monomer conversion (%MC) versus time are provided in Figures 1 and 2 and Tables S2 and S3. Upon polymerization of dimethacrylate monomers/macromers, C=C bonds are converted to C–C bonds, first joining all monomers together in a rapid free radical chain reaction. After 50% conversion, a slower cross-linking process takes over, leading to a three-dimensional network structure. Slowing of the reaction at 50% conversion was observed for all composite pastes (Figures 1 and 2). Reaction halts when the increasing monomer conversion raises the glass transition temperature of the paste above that of the surroundings, and the viscous paste rapidly changes into a glassy solid. At higher surrounding temperatures, greater final conversions and material glass transition temperatures are therefore expected. As shown in Figure 1, final composite conversions increased from ∼75% to over 90% on raising the temperature from 25 to 37 °C but were not significantly affected by changes in the filler.

Generally, the addition of BHT at 0.28 wt % (CP2, Figure 1) and 0.52–0.56 wt % (CP4–6, Figure 2) caused a delay of 2 and 4 min, respectively, but had minimal effect on subsequent reaction rate or final conversion. Delay being proportional to inhibitor concentration was as expected from kinetic theories.^35^ The delay provides enhanced injectability/working times of materials and was not significantly affected by temperature. An exception was observed when both HA and MCPM were included together, and pastes were investigated at 25 °C. In this case (CP7 and CP8, Figure 2), the delay was increased by 2–3 min. A possible explanation is that an increase in nonsilane-treated particles increases air incorporation. This can lead to enhanced oxygen inhibition of the polymerization process.

Previous similar experimental UDMA-based composites with low inhibitor levels possessed injectability times of less than 1 min and working times (at 50% MC) of less than 5 min,^5^ as seen with CP1 and CP3. The working times of commercially available bone cements are much longer (around 7–12 min).^36,37^ In this study, the BHT-added UDMA-based cements formulated with combined MCPM/HA (i.e., CP7 and CP8) had handling (injection) times (at 0% MC) of 5–6 min and working times (at 50% MC) of 8–11 min. After injection into the body, the cements are expected to reach the monomer conversions of approximately 90% that are observed at 37 °C. At this conversion, there are expected to be very few dimethacrylate molecules that have neither double bond reacted. Release of potentially cytotoxic uncured monomers is therefore unlikely.

### 3.2 Surface and Bulk Elemental Composition

**Figure 3:**
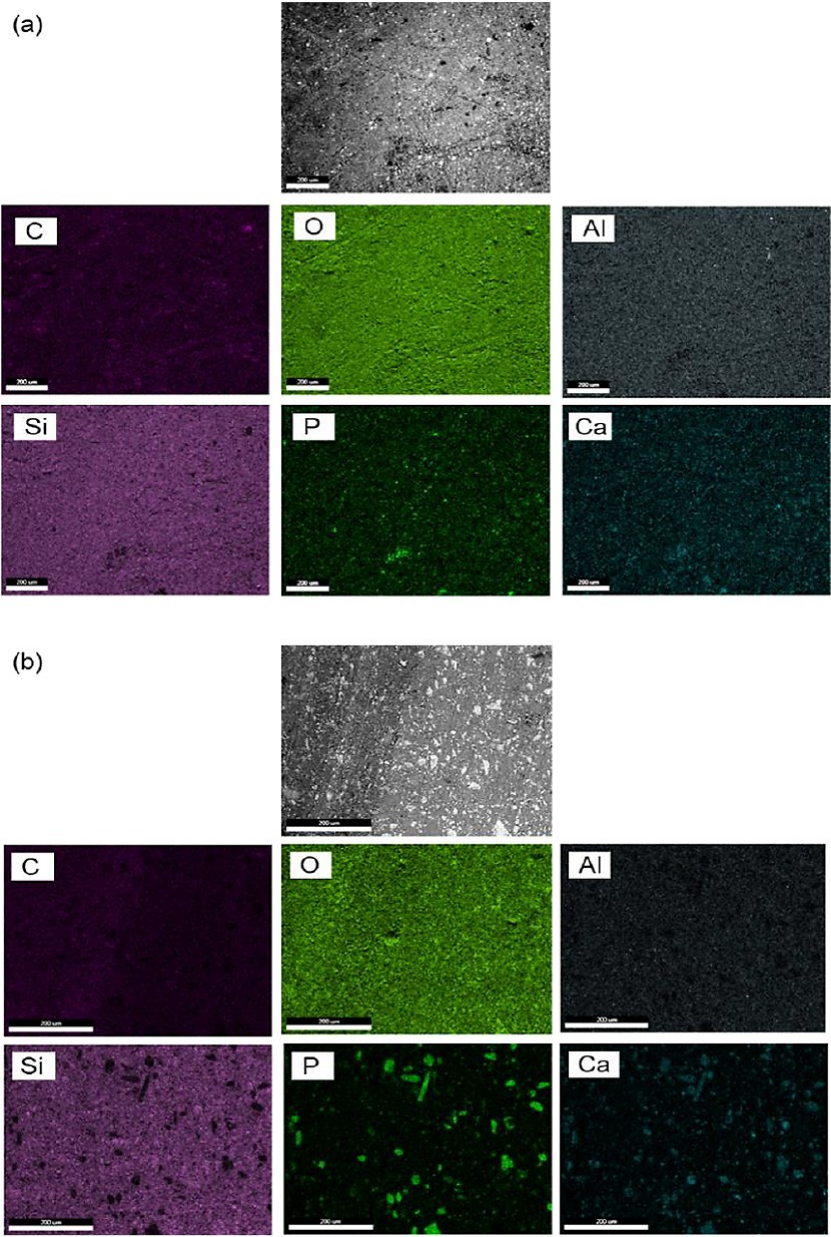
SEM images and corresponding EDX elemental mappings of the CP7 bone cement specimen’s surface (a) and cross-section (b).

To investigate the homogeneity of the particle distribution in the polymeric matrix of the bone cement, both the surface and cross-sectional areas of CP7 were examined by SEM/EDX analysis. Carbon, hydrogen, and oxygen are the main elements of the monomers, while oxygen, aluminum, and silicon are found in dental glasses. By weight, in HA and MCPM the ratio of calcium:phosphorus is 1:0.77 and 1:2.6, respectively. In both, the weight percentage of oxygen is approximately double the phosphorus content. Figure 3 reveals the SEM image and the corresponding EDX elemental mappings of carbon (C), oxygen (O), aluminum (Al), silicon (Si), phosphorus (P), and calcium (Ca) of the surface and cross-sectional region of the cement of CP7. More carbon, phosphorus, and calcium were detected in the fractured surface (Table 3). This was possibly due to the fracture line passing through the polymer phase and around HA and MCPM particle surfaces rather than at the silane-treated glass surfaces. The 50-μm MCPM particles gave bright regions in the phosphorus maps, less bright regions in the Ca maps and black holes in the Si map. Regions consistent with MCPM particles were only sporadically seen on the top surface. The smaller HA particles gave bright spots consistent with their size (7–20 μm) in both Ca and P maps due to their higher Ca:P ratio. More of these particles were detected on the cross-sectional surface (Table 3).

**Table 3:** Surface and Cross-Sectional Elemental Compositions of the CP7 Bone Cement Specimen Analyzed by SEM-EDX

elements	weight (%)	
	surface	cross-section
C	24.1 ± 2.0	35.5 ± 2.4
O	50.7 ± 0.7	39.4 ± 1.8
Al	3.9 ± 0.3	3.2 ± 0.3
Si	17.1 ± 1.9	14.4 ± 3.1
P	1.1 ± 0.3	2.4 ± 0.9
Ca	3.1 ± 0.6	5.1 ± 3.2

### 3.3 MSC Morphological Analysis

**Figure 4:**
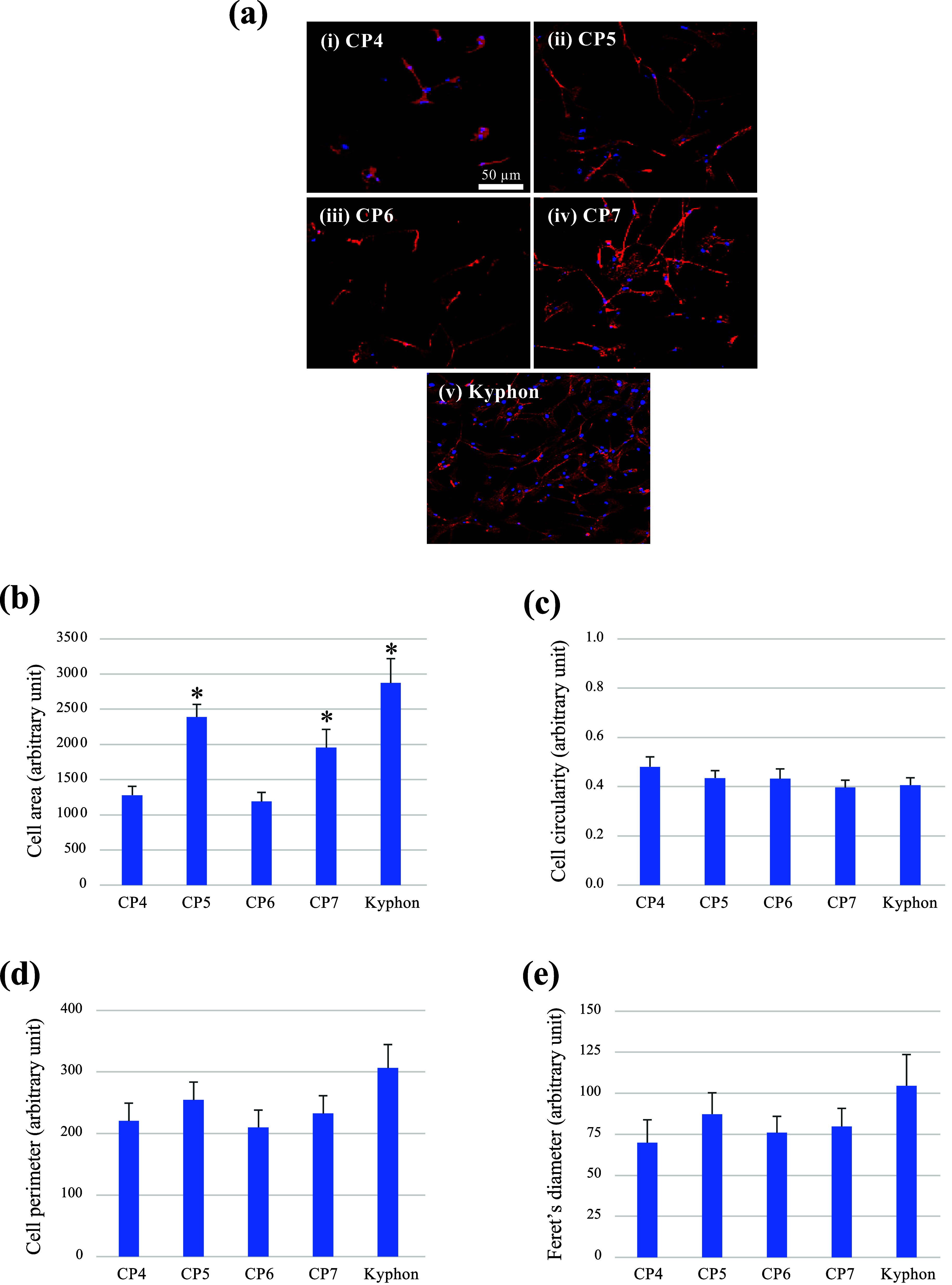
Morphological analysis of MSCs cultured on different composite bone cements for 24 h. Confocal immunofluorescence for actin filaments (red) and nuclei (blue) of cells grown on CP4 (i), CP5 (ii), CP6 (iii), CP7 (iv), and Kyphon (v) are shown in (a). The values of cell area, circularity, perimeter, and Feret’s diameter are comparatively plotted in (b–e), respectively. * *p* < 0.05 vs CP4.

Cell morphology is important in the evaluation of materials’ biocompatibility and osteoconductivity as it has been related to cell phenotype and state of differentiation.^38−40^ Cell spreading area, circularity, perimeter, and Feret’s diameter have been employed in the present study for the assessment of the morphological change of human MSCs grown on the different test materials, i.e., CP4 [CaP-free bone composite (coded as control composite)], CP5-CP7 (CaP-integrated bone composites), and Kyphon HV-R (high viscosity PMMA bone cement with 30% barium sulfate, Medtronic, USA) (coded as Kyphon). The results in Figure 4a demonstrated that growing adherent MSCs on the different surfaces showed slightly to moderately different morphology. Compared with star-like shaped MSCs on Kyphon, round/oval-shaped cells with short cellular processes were seen on the control composite (CP4). Conversely, spindle-shaped and polyhedral-shaped cells with large and long cellular processes were observed on the CaP-integrated bone composites (CP5-CP7), providing initial evidence that the CaPs used in this study influenced the morphology of MSCs. A summary of the analysis of cell area, circularity, perimeter, and Feret’s diameter is shown in Figure 4b–e; the average cell area values of MSCs seeded on all materials tested, except CP6, were significantly higher than that on CP4. In contrast, the circularity, perimeter, and Feret’s diameter of cells on all materials tested were not significantly different.

The influence of cell morphology and spreading area on osteogenic differentiation and maintenance of the differentiated phenotype of MSCs has previously been demonstrated; a larger cell spreading area promoted the osteogenic differentiation and maintenance of the differentiated phenotype in MSCs.^41^ Moreover, MSCs with star-like shapes and large areas more likely expressed genes associated with osteoblast differentiation and survival, compared to the small round MSCs with fewer cytoplasmic extensions.^39^ The qualitative cell shape and area analyses of MSCs on the composites containing either MCPM alone (CP5) or HA (CP7) and Kyphon were consistent with cell phenotypes related to high osteogenic potency and high survival rate, which might facilitate differentiation into mature osteoblasts immediately following the induction of osteogenic differentiation. However, the other cell morphology parameters, i.e*.,* cell circularity, perimeter, and Feret’s diameter, were not significantly influenced by the compositions of the cements tested, although these parameters have been associated with cell phenotype and state of differentiation.^38−40^ For example, pluripotent stem cells revealed a pronounced change from spherical to spindle-shaped cell morphology after differentiation induction, with their respective differentiating cells having a significantly higher Feret’s diameter and lower circularity.^42^

### 3.4 Growth of Human MSCs and Fibroblasts on Composite Bone Cements

**Figure 5:**
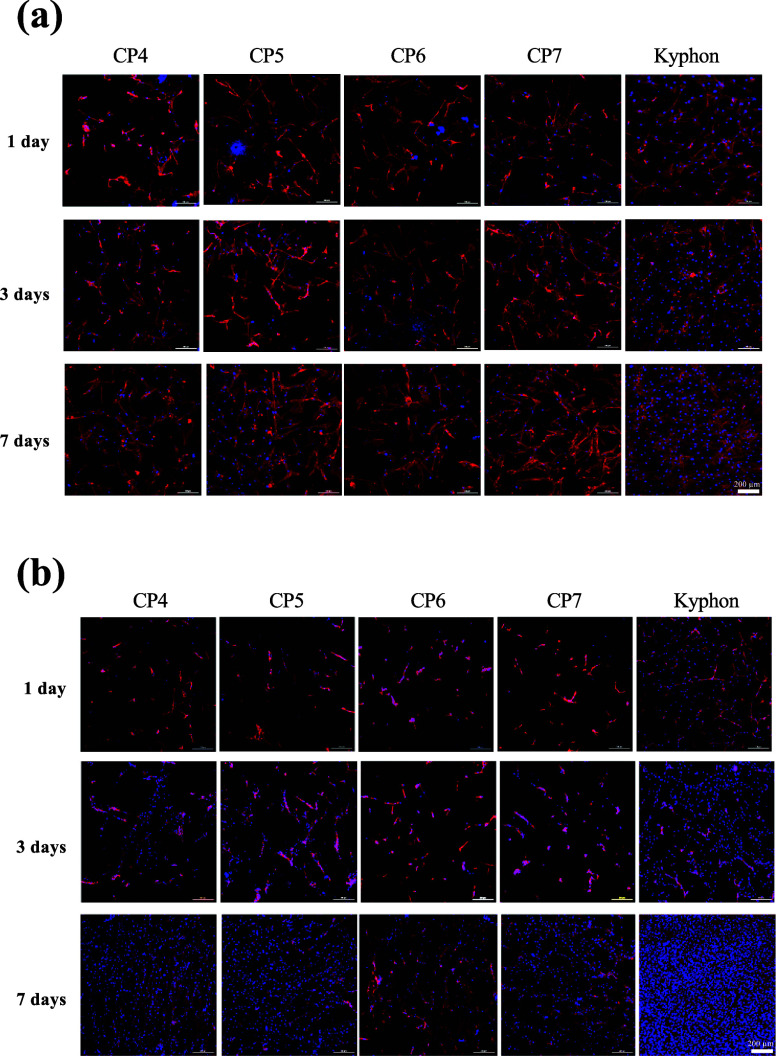
Morphology and growth of MSCs and fibroblasts on different bone cement groups. Confocal immunofluorescence for actin filaments (red) and nuclei (blue) of MSCs (a) and fibroblasts (b) cultured for 1, 3, and 7 days on the cement specimens are shown.

**Figure 6:**
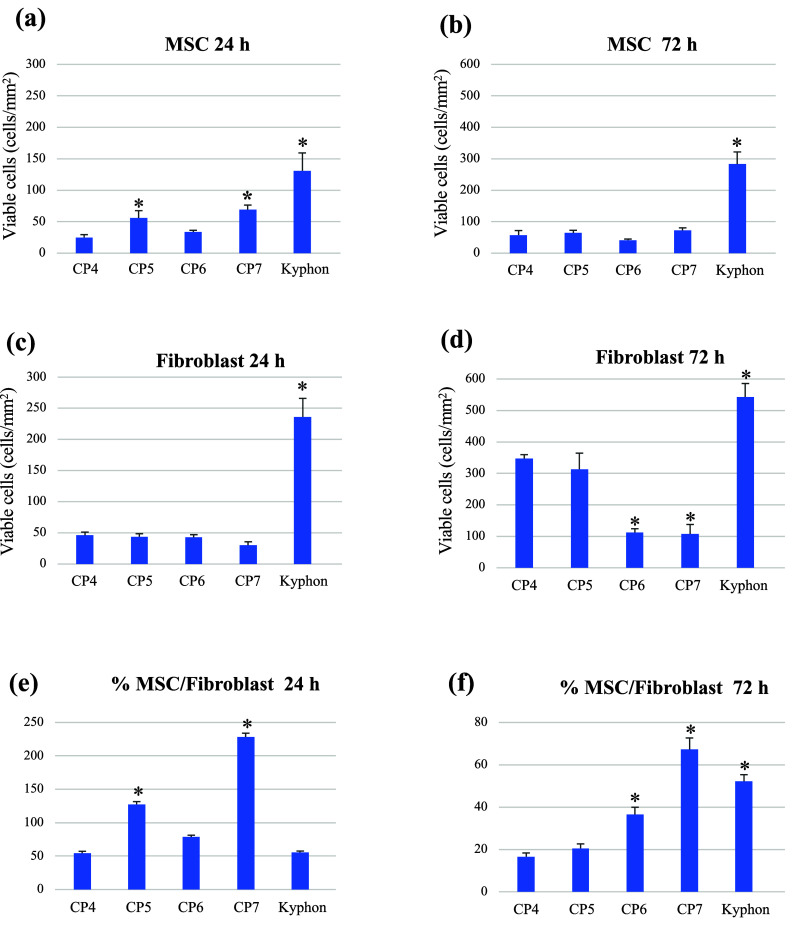
Quantification of the number of viable cells growing on different bone cement groups by the cell viability assay. The growth proportions of MSCs to fibroblasts at 24 and 72 h are also shown. * *p* < 0.05 vs CP4.

The results in Figure 5a demonstrated that MSCs grew slowly on CP4 (control composite without bioactive fillers) and CP6 (integrated with HA only) during the time studied; however, the addition of MCPM in CP5 and CP7 resulted in increased numbers of MSCs. Moreover, cells on MCPM-incorporated composites seemed to grow faster than those on the corresponding MCPM-free cements. The number of MSCs on Kyphon was higher than that of the experimental composites at all time points. Unlike MSCs, fibroblasts grew well on the control composite (CP4) and best on Kyphon throughout the tested time points (Figure 5b). Incorporating CaPs, especially HA, reduced the fibroblast number and growth rate. A summary of the quantitative analysis of the number of viable cells in Figure 6a,b shows a significantly higher number of viable MSCs cultured on the MCPM-added composites (CP5 and CP7) at 24 h only, but on Kyphon at both 24 and 72 h, compared with that observed on the control composite. Intriguingly, significantly lower numbers of fibroblasts were found on the HA-mixed composites (CP6 and CP7) (Figure 6c,d). Consequently, compared with that of the control composite, significantly greater ratios of numbers of viable MSCs to fibroblasts were perceived in the MCPM-integrated composites at 24 h but in the HA-incorporated composites and Kyphon at 72 h. Consequently, the MCPM and HA combined cement (CP7) showed the highest ratios at both time points (Figure 6e,f).

Taken together, the results suggested that MCPM could act as a growth-inducing mediator and directed toward an initial increase in the number of viable MSCs during the first 3 days in culture, and biomimetic HA probably helped prevent fibroblast growth later. Consequently, CP7 could effectively enhance the ratio of MSCs to fibroblasts at both 24 and 72 h, thereby promoting new bone formation while inhibiting fibrous tissue formation. It is noteworthy that the introduction of exogenous materials into the body frequently elicits a foreign body reaction, a multifaceted host response. This intricate process encompasses the initial adsorption of proteins onto the biomaterial surface followed by the recruitment and activation of inflammatory cells. Additionally, a foreign body reaction involves the activation of fibroblasts and the subsequent formation of a fibrous capsule surrounding the implanted material.^43^ It remains unclear how these two different forms of CaPs differentially regulate the growth of MSCs and fibroblasts. It has been reported that HA particles significantly decreased the number and proliferation of fibroblasts,^44,45^ plausibly by suppressing the protein production of transforming growth factor beta-1 (TGF-β1) and inducing the synthesis of prostaglandin E2 (PGE2).^44^ Further studies are needed to explore the in-depth mechanisms underlying this phenomenon.

### 3.5 Osteogenic and Mineralization-Inducing Activities of Composite Bone Cements

**Figure 7:**
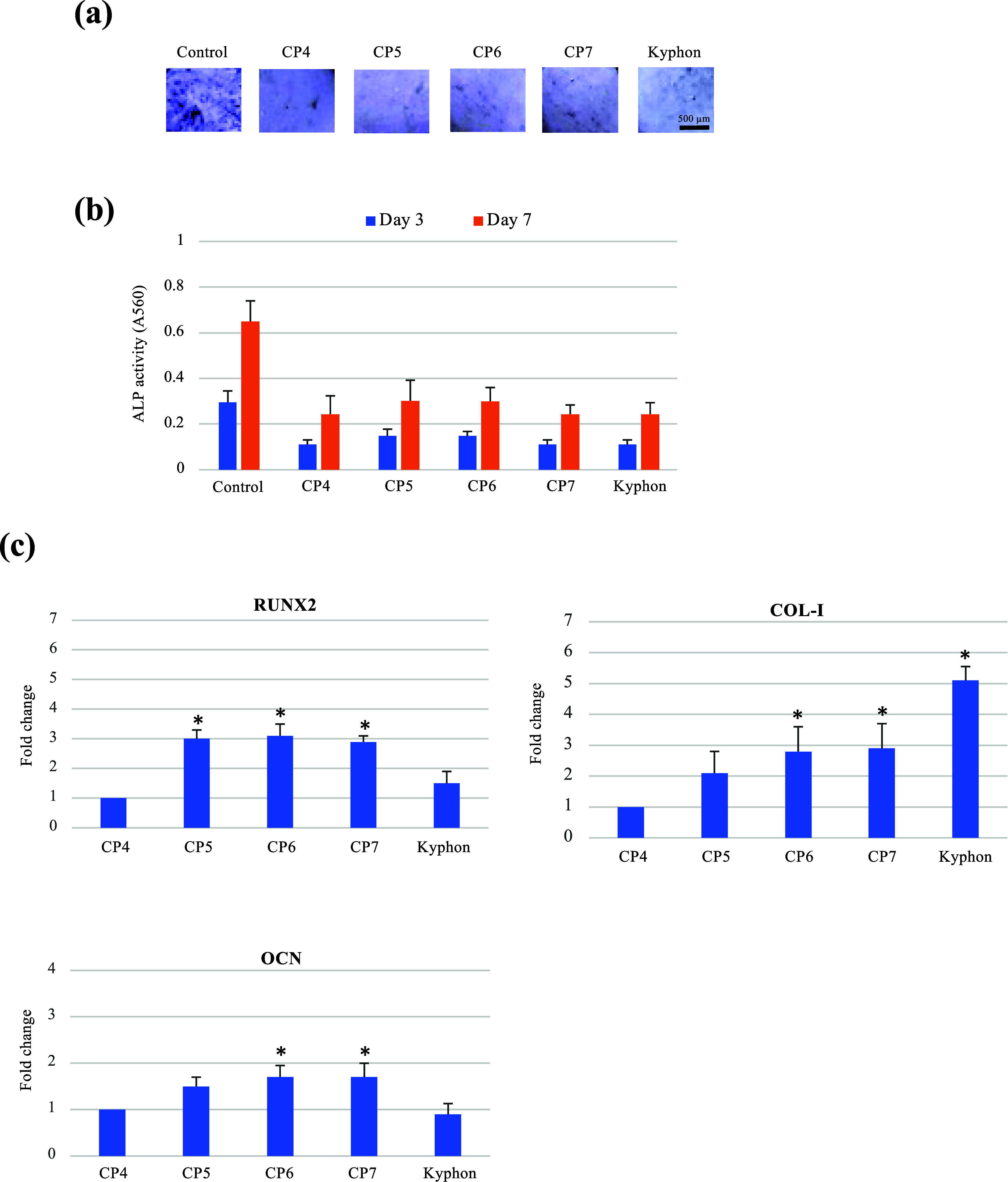
Osteoblast differentiation of MSCs cultured on different composite bone cements. MSCs were cultured on each cement under osteogenic induction, inducing MSCs to undergo osteoblast differentiation, and the histochemical analysis (a) and biochemical analysis (b) of the ALP activity and the relative expression of osteogenic genes (c) are shown. In (c), fold changes relative to those of the control group are summarized * *p* < 0.05 vs CP4.

Although lower than that detected with MSCs grown on the cell culture plate surface (control), all experimental composite cements (CP4-CP7) and Kyphon allowed the progression of ALP activity from Day 3 to Day 7 (Figure 7a,b). The presence of either HEMA or HA at a noncytotoxic concentration has previously been related to a decrease in ALP-specific activity of osteoblastic lineage cells.^45,46^ Opposite to the ALP activity, the expression of other osteogenic differentiation markers, i.e., RUNX2, COL-I, and OCN, was differentially upregulated by the composite cements and Kyphon (Figure 7c). The additions of the individual and combined CaPs in the composite cements consistently stimulated the expression of all the osteogenic genes studied, suggesting their osteogenic-inducing property. Noteworthily, the commercial PMMA cement had no stimulatory effect on the expression of RUNX2 and OCN genes, but showed a marked upregulation of COL-I gene expression, possibly explaining the formation of fibrous tissue in vivo.^47^

**Figure 8:**
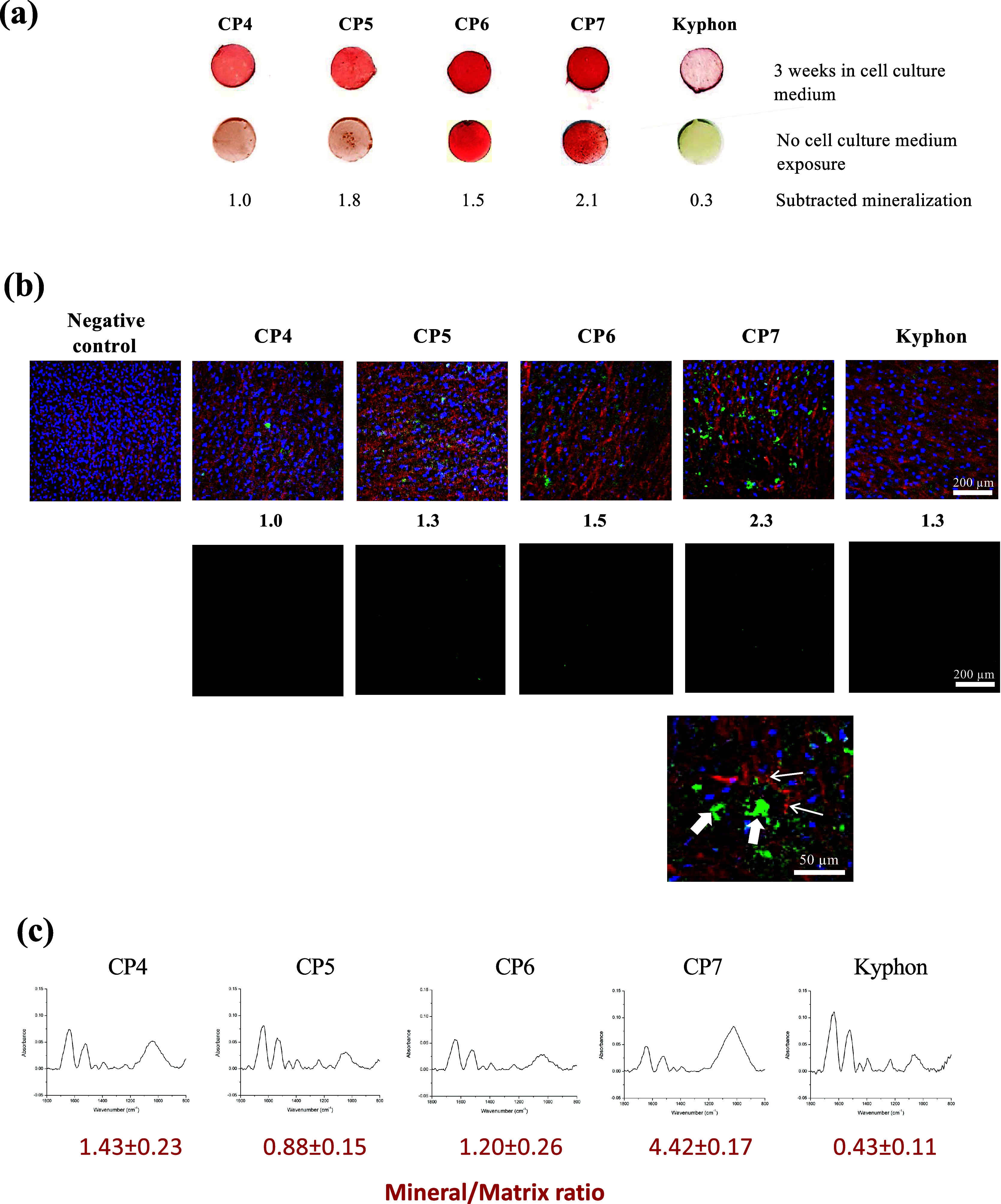
Effects of different composite bone cements on spontaneous and cell-induced mineralization. Individual cement discs were incubated in a cell culture medium for 3 weeks (with the medium being changed every 3 days). These and freshly prepared (control) samples were stained with alizarin red stained (a). Freshly prepared samples were also stained for background staining levels, which were used to subtract from those derived from samples exposed to the culture medium. The numbers indicating the relative positive staining, defined as 1.0 in the control composite, are also shown. In (b), confocal fluorescence images of tetracycline-stained samples for cell-induced mineralization observations are shown. MSCs were cultured on the specimens for 21 days under osteogenic induction, with two 24 h exposures of tetracycline. The numbers under the images show the relative levels of positive tetracycline staining (green), defined as 1.0 in the control CP4 composite. Background staining of each sample is also disclosed. A higher magnified fluorescence image of CP7 confirmed extracellular mineralization (thick arrows) and possible intracellular mineralization (thin arrows). In (c), MSCs were cultured on the composites for 21 days under osteogenic induction, and the cells (without specimens) were collected and processed for FTIR. Baseline-adjusted spectra derived from the individual samples are shown with the means ± SD of the mineral/matrix ratios. The mineral/matrix ratio values of swine spine cancellous bone (positive control) and MSCs cultured without OM (baseline control) are 5.8 and 0.2, respectively.

In the growth culture medium (having neither MSC seeding nor OM), composites containing MCPM or HA (CP7 and CP6) induced cell-independent spontaneous mineralization at 3 weeks (Figure 8a). The MCPM/HA-integrated cement (CP7) induced the most discernible mineralization by MSCs (Figure 8b). This cement increased the mineral/matrix ratio to a level 10-fold higher than that of the commercial cement (Figure 8c). In response to HA, a subset of osteogenic cells in MSCs might transiently dedifferentiate,^45^ leading to a decrease in ALP activity during the early period tested in this study (Day3–Day7 after osteogenic induction). However, subsequent exposure to HA-containing bone composites may trigger the redifferentiation of these cells along the osteoblastic lineage, potentially leading to a more committed phenotype upon the activation of master osteogenic transcriptional factors, such as RUNX2.^48^ This enhanced commitment may facilitate the transition of the osteoprogenitor cell to a preosteoblast state, characterized by the upregulation of markers most highly expressed during the differentiation of osteoblasts, such as ALP, COL-I, and OCN.^48^ Ultimately, these cells could differentiate into functionally mature osteoblasts, exhibiting an increased capacity for the production of mineralized deposits compared with those observed in non-HA-containing Kyphon. This evidently supported that CP7 could induce mineralization via spontaneous cell-independent and cell-mediated mechanisms, both of which produced mineralization higher than that formed on Kyphon.

### 3.6 Effects of Composite Bone Cements on the Growth of Osteoclast Precursors and Osteoclast Formation

**Figure 9:**
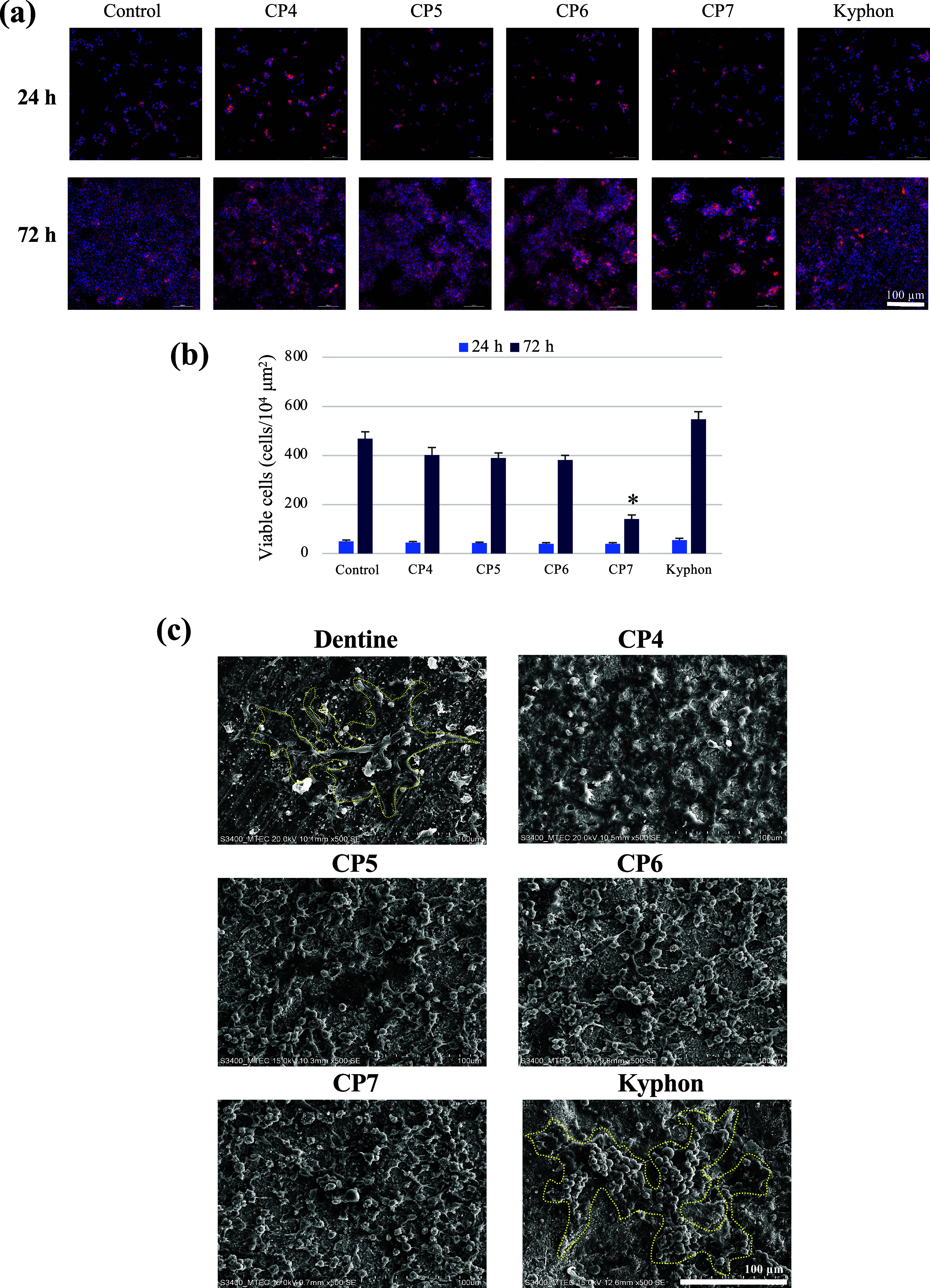

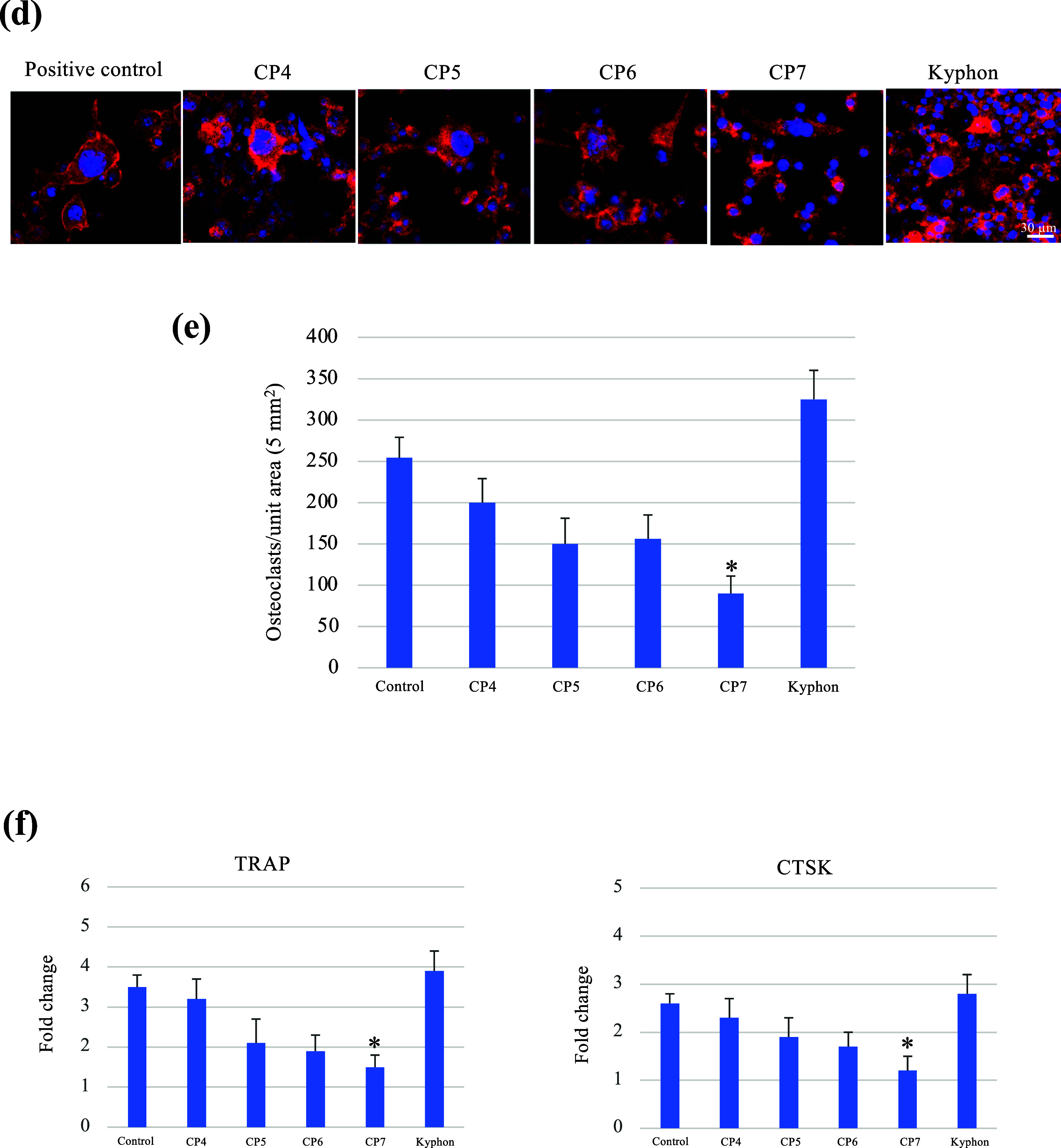
Growth of RAW cells on different composite bone cements and their effects on osteoclast formation. The cells were cultured on individual specimens for 24 and 72 h in the standard culture medium, and for visualization, they were stained for actin filaments (red) and nuclei (blue) (a). Cells cultured on the plastic well plate were used as a control. The quantification of viable cells after exposure to the materials was also carried out by using the cell viability assay (b). Under osteoclastic induction, RAW cells were cultured in an OCM for 4 days, followed by SEM analysis (c) and immunofluorescence staining for actin filaments (red) and nuclei (blue), and the formation of osteoclasts was evident by the presence of multinucleated giant cells having more than 2 nuclei (d). Yellow dashed lines in (c) help visualize the approximate boundary of osteoclasts formed on the dentine (as a positive control) and Kyphon. The numbers of osteoclasts/unit area are shown in (e). The expression of osteoclast-associated genes TRAP and CTSK on Day 3 following osteoclastic induction is shown in (f). * *p* < 0.05 vs control.

Figure 9a,b reveals a slight stimulation of growth of RAW cells from Day 1 to Day 3 when cells are cultured on Kyphon compared with those on the control plastic surface, and their growth was to some degree reduced by the cements formulated without CaPs and with either MCPM or HA. In contrast, the dual CaP-mixed composite cement markedly and significantly inhibited the growth of RAW cells by more than 250% (Figure 9b). The SEM and confocal immunofluorescence results demonstrated that fewer, smaller, multinucleated giant cells were observed in CP7, compared with those cultured on the other cement samples (Figure 9c,d, respectively). The number of osteoclasts formed on the CP7 surface was significantly inhibited by more than 2.5- and 2-fold compared with those found on positive control and CP4, respectively (Figure 9e). The reduced expression of TRAP and CTSK genes, which are associated with terminal osteoclast differentiation and maturation, further supported the inhibitory effect of CP7 on osteoclast formation (Figure 9f). The suppression of cellular fusion and osteoclast formation by the released calcium ions from MCPM and HA surface microstructure has previously been reported.^15,49^ Notedly, a lack of an osteoclast inhibitory role of Kyphon was observed in this study. The results suggested that MCPM and HA synergistically inhibited the formation of osteoclasts via the suppression of growth and differentiation of osteoclast precursor cells.

### 3.7 Differential Regulatory Role of MCPM/HA-Mixed Cement in PBMC Proliferation and Expression of Inflammation-Related Cytokine Genes

**Figure 10:**
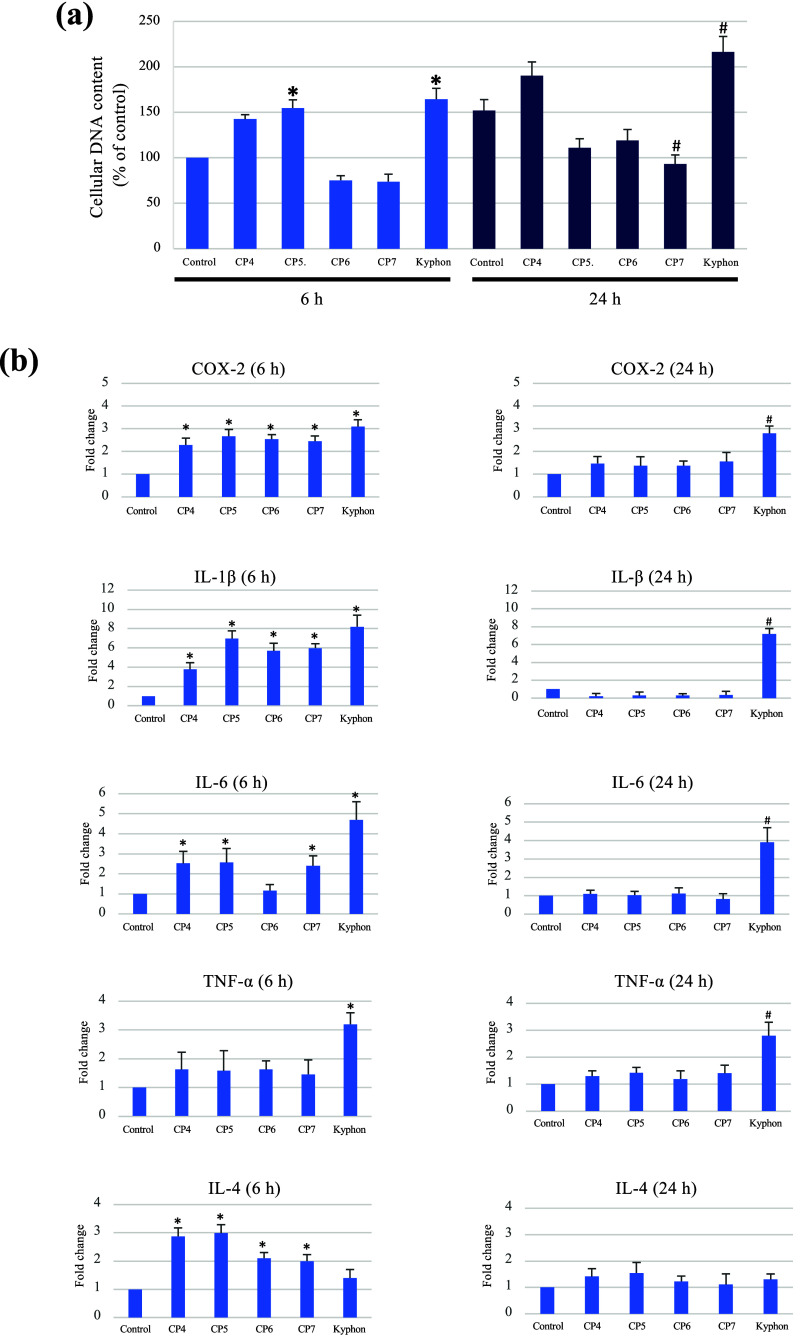
MCPM and HA differentially regulate proliferation and gene expression of key inflammation-related cytokines. In (a), LPS-induced PBMCs were cultured for 6 and 24 h on different composite bone cements, and the DNA contents were measured. The expression of COX-2, IL-1β, IL-6, TNF-α, and IL-4 at 6 and 24 h after cells being cultured on the individual specimens was examined by real-time qPCR (b). * *p* < 0.05 vs the control (cells cultured on the plastic well plate) at 6 h. ^#^*p* < 0.05 vs the control at 24 h.

Figure 10 shows that CP7 could suppress the proliferation of human PBMCs (Figure 10a) and initially stimulate proinflammatory cytokine gene expression, which subsequently decreased to a level similar to that of the control sample (Figure 10b). Unlike CP7, Kyphon increased the PBMC proliferation and the expression of all the cytokine genes studied, both at early and late time points investigated. The results also revealed that, in contrast to Kyphon, all the formulated composite cement samples upregulated the expression of the anti-inflammatory IL-4 gene (Figure 10b), suggesting that MCPM and HA differentially regulated the PBMC proliferation and cytokine gene expression, with HA being an active component as an immunoregulatory agent.

In the present study, two key cellular activation events, i.e., intracellular DNA content and the expression of cytokine, in PBMCs were investigated.^50^ The expression of crucial pro-inflammatory (COX-2, IL-1β, IL-6, and TNF-α) and anti-inflammatory (IL-4) genes was examined. Although MCPM rapidly and significantly activated PBMCs by increasing their cellular DNA content, HA inhibited their DNA content, enabling the MCPM/HA-incorporated cement not to overstimulate the PBMCs’ DNA content. It is possible that, in a cell culture medium, calcium and phosphate ions released from the highly reactive MCPM act as regulators for DNA synthesis, as previously reported,^51,52^ while the released calcium ions might be precipitated onto HA presented in the polymer matrix of CP7, resulting in no net increase in the concentration of free calcium and phosphate ions. Moreover, the biomimetic HA surface structure of microsized particles used in this study likely appeared not only nonreactive but also suppressive to PBMCs.^15^ IL-4 plays an important role in resolving inflammatory responses by inhibiting inflammation and possibly shifting the microenvironment from the inflammatory stage to the regeneration stage in the tissue healing process. The MCPM/HA-containing UDMA-based composite cement initially stimulated key cytokine gene expression by targeting such key inflammation-associated cytokines as COX-2, IL-1β, IL-6, and IL-4, but not TNF-α, by PBMCs, for a period of less than 24 h. Although the mechanism(s) by which CP7 regulated the gene expression of these cytokines remain(s) unclear from the present study, the responses appear to be favorable inflammatory responses that might not induce subsequent chronic inflammatory conditions, as might be observed in Kyphon. Such chronic inflammation is a well-known factor associated with fibrous encapsulation formed after PMMA implantation, which contains many mononuclear inflammatory cells and fibrous connective tissue.^53^ Future mechanistic studies of the immunomodulatory role of CP7 using suitable ex vivo and in vivo models specific for inflammatory bone destruction are needed. Well-characterized ex vivo and in vivo models of inflammatory bone destruction have previously been reported^54−56^ and are useful for the investigation of the therapeutic effects of the composite cements developed in this study.

## 4 Conclusions

This study reports the successful development of UDMA-based composite bone cements formulated with different cement compositions. The results demonstrated that varying BHT enabled a controllable delay before the reaction to provide time for injection. Subsequent rapid reaction and high final conversion (important to control cement placement and reduce potential long-term monomer release) were achieved. Set cements were nontoxic to osteo-immunological-related cell lineages studied, allowed sufficient MSC growth for subsequent mineralization, and limited the growth of fibroblasts, osteoclast progenitors, and PBMCs. Adding MCPM and/or HA to the cements differentially regulated in vitro MSC-derived osteoblast differentiation, cell-(in)dependent mineralization, osteoclast formation, and PBMS responses at cellular and molecular levels. This suggested their potential application in the reconstruction of inflammation-associated bone defects, which warrants further studies using ex vivo and in vivo models specific to inflammatory bone destruction.

## Supporting Information Available

Powder-to-liquid ratios of initiator and activator parts; FTIR spectra of the composite bone cement (CP7); and percentage monomer conversions (PDF)

## Notes

This study was performed in accordance with the principles of the Declaration of Helsinki. The Ethics Review Subcommittee for Research Involving Human Research Subjects of Thammasat University No. 3 (COA No. 068/2564) and the Institutional Biosafety Committee of Thammasat University (057/2564) granted approval.
