# Supplementary material for: In Vitro Osteo-Immunological Responses of Bioactive Calcium Phosphate-Containing Urethane Dimethacrylate-Based Composites: A Potential Alternative to Poly(methyl methacrylate) Bone Cement
Source: ACS Mater Au. 2024 Jul 18;4(6):612–27. doi: 10.1021/acsmaterialsau.4c00037 (PMC11565289; doi:10.1021/acsmaterialsau.4c00037)
Supplement: Supplementary file 1 — mg4c00037_si_001.pdf [file mg4c00037_si_001.pdf]

Supporting Information

Additional experimental details and supporting data related to the Results Section

In vitro osteo-immunological responses of bioactive calcium phosphate-containing urethane dimethacrylate-based composites: A potential alternative to poly(methyl methacrylate) bone cement

Weerachai Singhatanadgit[1], Piyarat Sungkhaphan[2], Boonlom Thavornyutikarn[2], Setthawut Kitpakornsanti[1], Anne M Young[3*], Wanida Janvikul[2*]

[1]Faculty of Dentistry and Research Unit in Mineralized Tissue Reconstruction, Thammasat University (Rangsit Campus), Thailand

[2]National Metal and Materials Technology Center, National Science and Technology Development Agency, Thailand

[3]Division of Biomaterials & Tissue Engineering, UCL Eastman Dental Institute, Royal Free Hospital, Hampstead, London NW3 2PF, UK.

*Authors for correspondence

Additional experimental details

Table S1. Powder-to-liquid ratios of initiator and activator parts of the formulated cement pastes.

Composite pastes	Initiator parts (wt%)			Activator parts (wt%)		
	Monomers*	Powders**	P/L ratios	Monomers*	Powders**	P/L ratios
CP1	27.54	71.59	2.6:1	27.63	71.85	2.6:1
CP2	24.94	67.20	2.6:1	24.59	65.26	2.6:1
CP3	24.43	74.82	3:1	24.50	75.00	3:1
CP4	24.01	74.99	3:1	24.50	75.00	3:1
CP5	24.01	74.99	3:1	24.50	75.00	3:1
CP6	29.07	69.71	2.3:1	25.13	74.36	3:1
CP7	29.09	69.68	2.3:1	25.14	74.35	3:1
CP8	29.24	69.70	2.3:1	25.13	74.36	3:1

Supporting data related to the Results Section

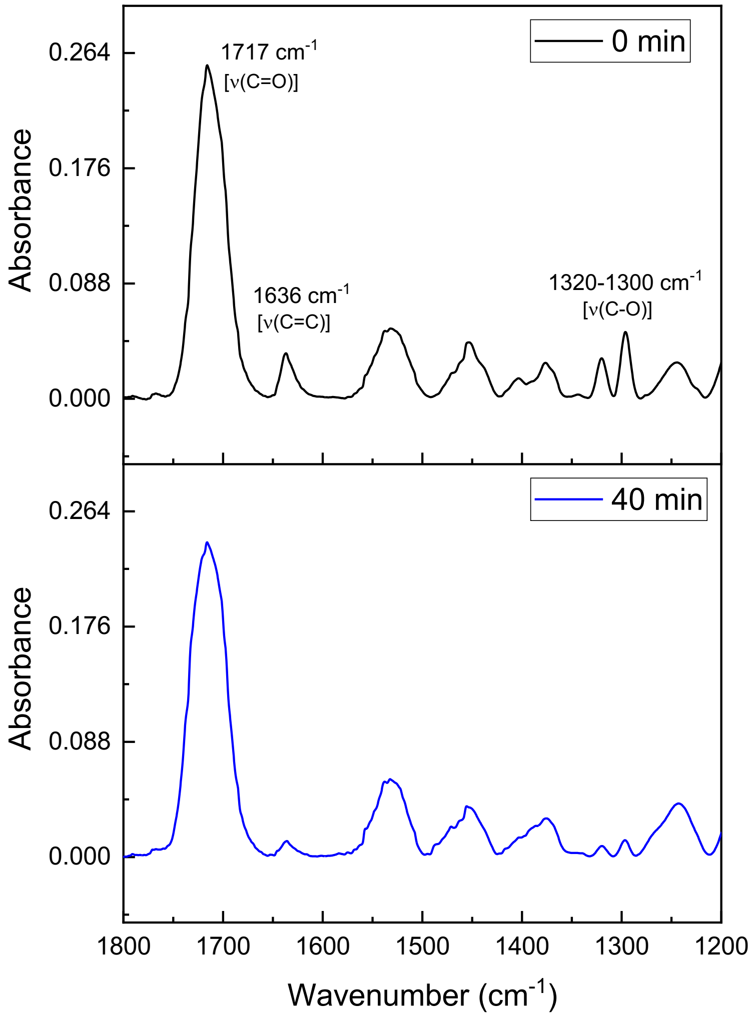

Figure S1. FTIR spectra of the composite bone cement (CP7) recorded at 0 and 40 min.

Table S2. Percentage monomer conversions as a function of polymerization time of CP1-CP2, determined from their ATR-FTIR spectra continuously acquired at 25°C and/or 37°C for 40 min.

Polymerization times (min)	Monomer conversions (%)		
	CP1 (37°C)	CP2 (25°C)	CP2 (37°C)
0	0	0	0
0.4	7	0	0
1	36	0	0
2	60	0	1
3	68	28	44
4	71	46	57
5	74	49	64
6	77	56	70
7	78	60	72
8	79	60	76
9	82	64	76
10	81	64	79
15	85	70	84
20	86	73	84
25	89	73	88
30	90	73	90
35	90	77	92
40	91	77	93

Table S3. Percentage monomer conversions as a function of the polymerization time of each formulated composite paste, determined from the ATR-FTIR spectra continuously acquired at 25°C for 40 min.

Polymerization times (min)	Monomer conversions (%)					
	CP3	CP4	CP5	CP6	CP7	CP8
0	0	0	0	0	0	0
0.4	0	0	0	0	0	0
1	5.5±2.1	0	0	0	0	0
2	25.0±7.1	0	0	0	0	0
3	36.5±9.2	0	0	0	0	0
4	46.5±6.4	0	0	0	0	0
5	50.5±6.4	9.1±2.0	10.7±3.6	5.2±1.7	0	0
6	54.0±5.7	29.9±0.5	31.7±1.8	28.9±3.4	0	14.8±0.9
7	57.5±6.4	40.2±0.7	42.8±2.3	43.0±2.8	12.2±2.7	40.7±0.4
8	60.5±6.4	48.7±2.5	49.7±2.2	48.5±1.6	19.0±3.4	51.9±5.2
9	60.5±6.4	52.1±0.8	53.6±1.7	53.5±0.0	33.0±2.9	55.4±4.9
10	62.5±3.5	55.5±0.8	57.1±1.7	56.8±0.0	42.1±4.6	59.2±4.8
11	60.5±6.4	59.0±2.7	58.8±0.0	58.5±1.6	50.3±4.4	59.4±4.9
12	60.5±6.4	60.7±1.0	60.5±1.7	61.8±1.6	56.5±4.4	60.4±5.6
13	60.5±6.4	62.4±0.7	62.2±0.0	61.8±1.6	57.8±3.6	62.9±4.6
14	62.5±3.5	62.4±0.7	62.2±0.0	61.8±1.6	58.8±2.8	65.2±3.4
15	66.0±4.2	62.4±0.7	65.7±0.0	63.5±0.0	61.9±2.8	65.2±3.4
20	69.0±4.2	69.5±0.2	69.1±0.0	70.1±0.0	67.4±1.5	68.7±3.0
25	73.0±4.2	71.2±1.5	69.1±0.0	71.8±1.7	71.7±1.2	72.2±2.7
30	75.0±1.4	71.2±1.5	70.8±1.7	73.4±0.0	72.7±0.2	72.2±2.7
35	75.0±1.4	72.9±0.2	74.2±1.7	75.1±1.7	73.7±1.3	74.4±4.2
40	77.0±0.0	72.9±0.2	74.2±1.7	75.1±1.7	74.7±1.6	75.7±2.4
